# And Littler Genomes inside 'Em: Clues to a Parasitic Nematode's Bacterial Partnership

**DOI:** 10.1371/journal.pbio.0030148

**Published:** 2005-03-29

*“Great fleas have little fleas upon their backs, to bite 'em. And little fleas have lesser fleas and so on,* ad infinitum.”—*Augustus DeMorgan, based on a Jonathan Swift poem*

More than a billion people are at risk for infection with filarial nematodes, parasites that cause elephantiasis, African river blindness, and other debilitating diseases in more than 150 million people worldwide. The nematodes themselves play host to bacteria that live within their cells, but in this case, the relationship is classic mutualism, with each benefiting from the other. Indeed, the Wolbachia bacterium is so crucial to its host nematode that apparently eradicating it with antibiotics severely compromises the nematode's ability to complete its life cycle within its human host. Thus, understanding the details of this symbiosis may help identify new strategies for controlling diseases caused by filarial nematodes. In a new study, Barton Slatko and colleagues present the complete DNA sequence of the Wolbachia pipientis strain within Brugia malayi, a parasitic nematode responsible for lymphatic filariasis, and analyze its genome for clues to the interdependence of the two species.

This Wolbachia genome is small, only about a million base pairs, and many metabolically critical genes have degraded through mutation to the point of uselessness. This phenomenon, called reductive evolution, is typical of long-term symbioses, as the two partners increasingly complement one another's biochemical activities, reducing the selection pressure on otherwise lethal mutations. Wolbachia's translational machinery and DNA repair equipment are largely intact. The bacterium appears to supply nucleotides to its host, as it contains complete pathways for biosynthesis of both purine and pyrimidine nucleotides. This is in contrast to Rickettsia, a close relative of Wolbachia and a mammalian parasite. Slatko and colleagues enumerate a variety of other pathways that have either been degraded or preserved, and highlight patterns in the genome structure through comparisons with both Rickettsia and another Wolbachia strain, found in fruit flies. For example, the two Wolbachia strains appear to have different membrane structures, possibly reflecting their different lifestyles (mutualistic versus parasitic).[Fig pbio-0030148-g001]

**Figure pbio-0030148-g001:**
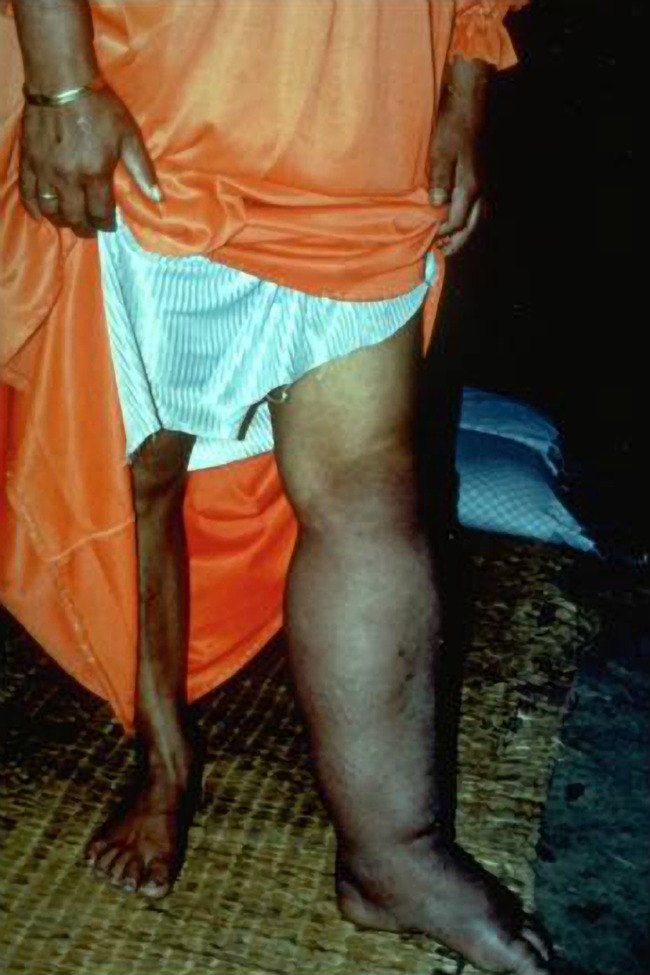
Over a billion people are at risk for infection by filarial nematodes, parasites that cause elephantiasis (Photo: Dr. Steven A. Williams)

Wolbachia can manufacture riboflavin and FAD, which are essential metabolic coenzymes and which do not appear to be made by its host. Conversely, it cannot synthesize amino acids and a variety of other vitamins and cofactors, and probably depends on the nematode to supply them. One discovery of possible significance is the presence in the bacterium of the synthetic pathway for heme—the oxygen-carrying iron component of hemoglobin. The nematode may require heme for synthesis of developmental hormones, so Wolbachia's heme pathway may be an inviting target for therapy against nematode infection. Since no new antifilarial has been developed in two decades, these results may quickly lead to new therapeutic strategies against these parasites.

